# *Candida parapsilosis* Characterization in an Outbreak Setting

**DOI:** 10.3201/eid1006.030873

**Published:** 2004-06

**Authors:** Duncan M. Kuhn, Pranab K. Mukherjee, Thomas A. Clark, Claude Pujol, Jyotsna Chandra, Rana A. Hajjeh, David W. Warnock, David R. Soll, Mahmoud A. Ghannoum

**Affiliations:** * University Hospitals of Cleveland and Case Western Reserve University, Cleveland, Ohio, USA;; † Centers for Disease Control and Prevention, Atlanta, Georgia, USA;; ‡ University of Iowa, Iowa City, Iowa, USA

**Keywords:** *Candida parapsilosis*, biofilm, adherence, secreted aspartyl proteases, phospholipase, outbreak, virulence

## Abstract

*Candida parapsilosis* is an important non-*albicans* species which infects hospitalized patients. No studies have correlated outbreak infections of *C. parapsilosis* with multiple virulence factors. We used DNA fingerprinting to determine genetic variability among isolates from a *C. parapsilosis* outbreak and from our clinical database. We compared phenotypic markers of pathogenesis, including adherence, biofilm formation, and protein secretion (secretory aspartic protease [SAP] and phospholipase). Adherence was measured as colony counts on silicone elastomer disks immersed in agar. Biofilms formed on disks were quantified by dry weight. SAP expression was measured by hydrolysis of bovine albumin; a colorimetric assay was used to quantitate phospholipase. DNA fingerprinting indicated that the outbreak isolates were clonal and genetically distinct from our database. Biofilm expression by the outbreak clone was greater than that of sporadic isolates (p < 0.0005). Adherence and protein secretion did not correlate with strain pathogenicity. These results suggest that biofilm production plays a role in *C. parapsilosis* outbreaks.

The yeast *Candida* is the fourth most common cause of hospital-related bloodstream infections (*1*). Forty percent of patients who have had *Candida* isolated from their intravenous catheters have underlying fungemia (*2*), and the case-fatality rate for catheter-related candidemia approaches 40% (*3*).

Although *C. albicans* is the most commonly isolated yeast, other species are found with increasing frequency, including *C. parapsilosis* (*4*). *C. parapsilosis* particularly affects critically ill neonates and surgical intensive care unit (ICU) patients (*5**,**6*), likely because of its association with parenteral nutrition and central lines (*7**,**8*). The affinity of *C. parapsilosis* for foreign material is shown by infections related to peritoneal dialysis catheters (*9*) and prosthetic heart valves (*10*), and this characteristic may be important in infections of cancer patients with indwelling access devices (*11*). *C. parapsilosis* is increasingly responsible for hospital outbreaks, and the hands of healthcare workers may be the predominant environmental source (*12*).

Our understanding of fungal virulence factors is limited. The surface adherence capacity of *Candida* is likely one such factor, possibly linked to its subsequent ability to form biofilms (*13*). Clinically obtained *C. albicans* isolates form biofilms (*14*), which may be important for sustaining infection. Adhesion (*15*) and biofilm formation (*14*) may be especially important for *C. parapsilosis*, since indwelling devices appear to be the predominant route of infection (*8**,**11*). Total parenteral nutrition (TPN) solutions may promote *C. parapsilosis* adhesion and growth (*16*). Recently, biofilm-forming potential was cited as a reason that patients with *C. parapsilosis*-infected catheters should have the device removed (*17*).

Fungi secrete enzymes integral to pathogenesis. Phospholipases (e.g., phospholipase B) and proteases (e.g., secreted aspartyl proteases [SAPs]) are two of the best-characterized. Although phospholipase B expression has been well studied in *C. albicans* (*18*), the relationship between *C. parapsilosis* virulence and phospholipase phenotype is unclear. The role of SAP and pathogenesis is similarly unclear (*19*).

We characterized genetic and phenotypic characteristics of isolates from a *C. parapsilosis* outbreak that occurred in a Mississippi community hospital (*20*) and compared these characteristics with those of isolates obtained from persons with sporadic infections at our tertiary hospital. We performed molecular characterization, comparing *C. parapsilosis* isolates involved in the outbreak with those from our own clinical collection. We then compared adhesion ability, biofilm production, and secretion of SAP and phospholipase B of the outbreak isolates and our clinical strains.

## Methods

### Organisms

Outbreak isolates of *C. parapsilosis* were obtained at a Mississippi hospital from April through October 2001. Epidemiologic details regarding the organisms and patients have been published elsewhere (*21*). We examined five invasive strains (defined as obtained from blood or catheter cultures: 165, 167, 173, 177, 179), and three environmental isolates (from healthcare workers' hands; 313, 317, and 385). Isolates from sporadic infections were from the culture collection at the Center for Medical Mycology, University Hospitals of Cleveland (University Hospitals). *C. parapsilosis* strain P/A71 was obtained from sputum, P92 from blood, and *C. albicans* M61 from an intravascular line (*19*). The site of isolation of other strains is indicated in the figures. Speciation was performed by using germ tube tests and API20C-AUX methods. Organism propagation has been described previously (*21*). All specimens were stored and used without patient identifiers, to maintain confidentiality.

### DNA Fingerprinting

Isolates were analyzed by Southern blot hybridization using the complex DNA fingerprinting probe Cp3-13 (*22*), according to published methods (*23*). Genomic DNA was extracted from cells according to the protocol described by Scherer and Stevens (*24*). Three micrograms of DNA preparation were digested with a combination of *Eco*RI and *Sal*I (4 U each per microgram of DNA) for 16 h at 37°C, then underwent electrophoresis at 60 V in a 0.7% agarose gel. *C. parapsilosis* strain J940043 was used as a reference, and its DNA was run in the first and last lanes of the fingerprinting gel. The DNA was transferred from the gel to a nylon Hybond N^+^ membrane (Amersham, Piscataway, NJ) by capillary blotting, prehybridized with sheared salmon sperm DNA, hybridized overnight with [^32^P]dCTP-labeled Cp3-13 probe, and viewed by autoradiogram.

### Computer-Assisted Cluster Analysis

The autoradiogram image was digitized, unwarped, and straightened, by using the DENDRON software database (*25*). Processed hybridization patterns were scanned to identify and link common bands. Patterns underwent pairwise comparison: the similarity coefficient (S_AB_) between the patterns of every pair of isolates A and B was computed according to the formula:

*S*_AB_=*2E/(2E + a + b)*

where *E* is the number of bands common to both strains, *a* is the number of bands unique to strain A, and *b* is the number of bands unique to strain B (*22*). The *S*_AB_ ranges from 0.0 (no common bands) to 1.0 (identical match of all bands). Dendrograms based on *S*_AB_ values were generated by the unweighted pair-group method with arithmetic average (UPGMA) (*26*); values of 0.07 were considered the threshold for group association (*27*).

### Adherence Assays

Adherence of *C. parapsilosis* isolates to silicon elastomer (SE) disks was measured by using a modification of earlier methods (*28*); SE was obtained from Cardiovascular Instrument Corp. (Wakefield, MA) and prepared as described (*14*). Standardized suspensions of 50 to 200 cells/mL were used for injection into SE disks. After injection, disks were washed in phosphate-buffered saline (PBS) to remove nonadherent cells and placed in wells of 12-well tissue culture plates (Becton Dickinson, Franklin Lakes, NJ). Two milliliters of warm (55°C) liquid SD agar was added per well to completely cover the SE disks and allowed to solidify. Plates were incubated overnight (37°C), and colonies adhering per disk were counted by using a dissecting microscope.

### Biofilm Formation and Quantitation

*C. parapsilosis* biofilms were formed on SE disks as described previously (*14*). Control disks were handled identically, except that no blastospores were added. Biofilm quantitation was performed as described (*21*) with dry weight measurements. Dry weight measured total biofilm mass including fungal cells and extracellular matrix.

### SAP Assays

Previous authors have described methods of evaluating the ability of *Candida* SAP to degrade bovine serum albumen (BSA) from cells grown in SAP expression media (*29*). We grew *C. parapsilosis* isolates in yeast nitrogen base (YNB) because we could detect SAP activity using YNB, the use of expression medium resulted in contaminating BSA bands, and our assay utilized the same medium used for examining biofilm formation. To confirm relevance of our findings to those of previous studies, we examined SAP expression of organisms grown in the expression medium and found similar results (data not shown).

After overnight growth, *Candida* cell suspensions were centrifuged (6000 *g* for 8 min), and the supernatant was collected, then concentrated by using a Centricon 10,000 NMWL filter centrifuge (Millipore Corp., Billerica, MA). Supernatant protein (500 ng) was incubated at 37°C for 15 min with 0.4 mL of 1% BSA (wt/vol, in 0.1 mol/L citrate buffer, pH 3.2). After incubation, 10 µL sodium dodecyl sulfate (SDS) sample buffer and 7 µL of reducing agent were added to 40 µL of each mixture, and the proteins solubilized by boiling (10 min). Ten microliters of sample was separated by SDS–polyacrylamide gel electrophoresis (PAGE), and the protein bands were visualized by silver staining (SilverXpress Staining Kit, Invitrogen Corp., Carlsbad, CA). The appearance of a 20-kDa band was indicative of SAP activity. Quantitation of this band was determined by using QuantOne software v4.3.0 (BioRad Laboratories, Hercules, CA). Control experiments were performed by adding either no supernatant (100 µL of sodium citrate buffer instead) or supernatant mixed with protease inhibitor cocktail (Sigma Chemical Co., St. Louis, MO); 10 µL/mL of supernatant). Protein estimations were performed by using the BioRad D*c* kit (BioRad Laboratories) and BSA as standard.

### Phospholipase Assays

A colorimetric assay for free fatty acid (FFA) was used to assess phospholipase activity (*30*). The incubation mixture for phospholipase (acylhydrolase) activity consisted of 200 µM dipalmitoyl (C16:0) phosphatidylcholine and 200 µmol/L L-palmitoylcarnitine in 0.1% (vol/vol) Triton X-100. Concentrated culture supernatant was added (100 µg of total protein), and the mixture made up to a final volume of 0.25 mL with 0.1 mol/L of sodium citrate, pH 4.0. Reactions were incubated at 37°C for 1 h, then stopped by adding chloroform/methanol (1:2, vol/vol). The reaction products were extracted (*31*), evaporated to dryness under nitrogen, and taken up in 50 µL of 0.1% (vol/vol) Triton X-100. The relative level of free fatty acids in each sample was determined by using an acyl-CoA-oxidase system assay kit (Roche Molecular Biochemicals, Indianapolis, IN).

### Statistical Analysis

Adherence and biofilm experiments were performed in quadruplicate and on separate days. Results for different isolates were normalized to *C. parapsilosis* strain 167 to facilitate meaningful comparisons across multiple experiments (*14*). Phospholipase and SAP assays were performed at least twice; representative results are shown. Statistical analysis was performed by using StatView v5.0.1 software (SAS Institute, Cary, NC); p values < 0.05 were considered significant.

## Results

### DNA Fingerprinting Analysis

**Figure 1:**
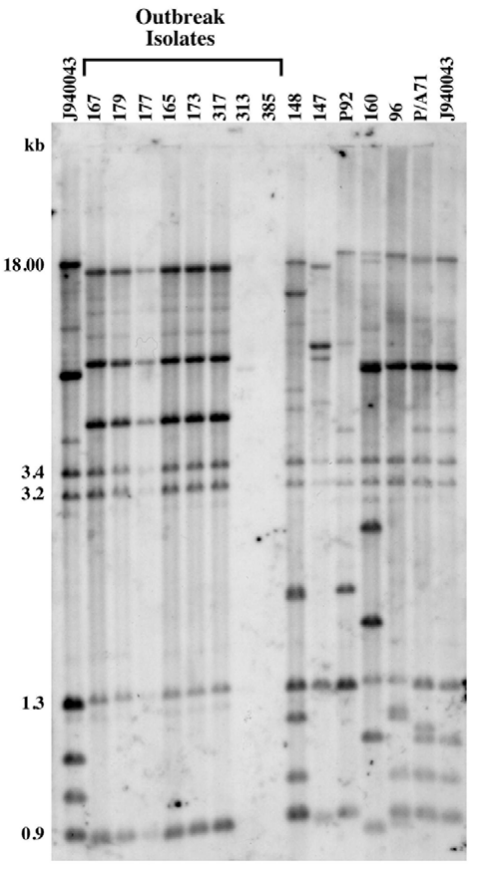
Genetic analysis of *Candida parapsilosis* clinical isolates. Southern blot hybridization patterns of the 14 *C. parapsilosis* test isolates were probed with the Cp3-13 DNA fingerprinting probe. The reference strain J940043 was run in the outer two lanes of the gel. Isolates associated with the hospital outbreak are indicated. Note that while isolates 167, 179, 177, 165, 173, and 317 displayed identical group I patterns, strains 313 and 385 showed patterns typical of non-group I strains with a lack of abundant intense bands. Molecular sizes are presented in kilobases to the left of the panel.

Isolate relatedness was investigated by using the complex DNA fingerprinting probe Cp3-13 (*22*). We examined both outbreak strains and our independent University Hospitals' isolates to characterize the relatedness of a range of clinical *C. parapsilosis* strains. As shown in Figure 1, the five invasive strains and one of the three environmental isolates generated identical patterns. The two remaining strains (313 and 385) were hand isolates. The fingerprinting pattern of strain 313 was limited to weak bands, while none were obtained for 385. The patterns of the outbreak isolates were also distinct from those of the University Hospitals' isolates.

**Figure 2:**
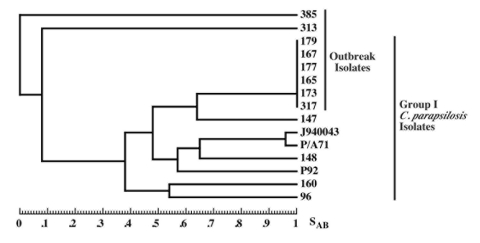
Relatedness of *Candida parapsilosis* clinical isolates. Dendrogram generated from S_AB_s computed for pairwise comparisons of the 14 *C. parapsilosis* test isolates and the reference strain J940043 fingerprinted with Cp3-13. Note that with the exception of the six identical outbreak isolates, none of the dendrogram nodes exceed an S_AB_ value of 0.7 for the other test isolates.

Relatedness among outbreak and University Hospitals' isolates was further assessed through cluster analysis (Figure 2). The six outbreak isolates with an identical fingerprinting pattern had an *S*_AB_ value close to 1, whereas the dendrogram nodes linking the remaining isolates to each other had an *S*_AB_ value <0.7, the threshold for relatedness (*27*). These analyses showed that the six outbreak isolates were identical and belonged to group I strains (*22*). The remaining isolates appeared moderately related to unrelated at the genetic level, and therefore were non–group I strains. Previous studies have shown that Cp3-13 fingerprinting patterns made up of a few weak bands typically belong to groups II or III, representing a minority of *C. parapsilosis* clinical isolates (*22**,**32*). However, internally transcribed spacer region sequencing of strain 313 indicates that it, in fact, belongs to group 1 (D. Warnock, pers. comm.). Alternately, this finding may suggest past genetic exchanges between group I and non–group I strains. The dendrogram also shows that, in addition to being unrelated to the outbreak isolates, the University Hospitals' strains are not related to one another but represent sporadic cases.

### Adherence

**Figure 3:**
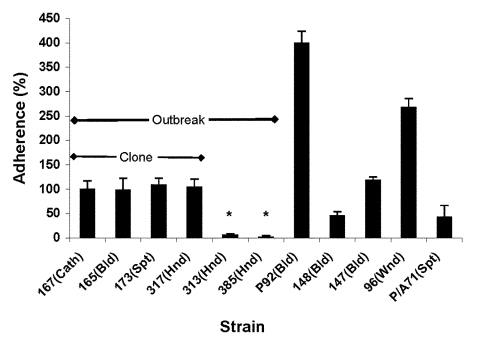
Adherence properties of *Candida parapsilosis* clinical isolates. Graph shows adhesion ability of various *C. parapsilosis* strains, compared to strain 167 from the Centers for Disease Control and Prevention. Results were normalized to strain 167, which was taken as 100%. Each result is representative of at least two experiments. Error bars represent standard deviation. *p < 0.001 for comparison of values of strain 167 vs. strains 313 and 385; all other comparisons had p values > 0.05. (For details of methods used, see text.) Cath, catheter; Bld, bloodstream; Spt, sputum; Hnd, hand; Wnd, wound; Pdf, peritoneal dialysis fluid.

Adherence to substrate, whether natural (endothelium) or artificial (catheter material), is likely the first step in *Candida* pathogenesis (*33*). As shown in Figure 3, the adherence abilities of *C. parapsilosis* isolates vary widely. Adherence was the same for outbreak isolates of the same clone (167, 165, 173, 317; other clonal isolates were excluded for clarity), regardless of the site of isolation. Adherence was significantly higher than for the two unrelated hand isolates (313 and 385; p < 0.001). No relationship was found between the outbreak isolates and University Hospitals' isolates, although the latter exhibited higher values than strain 313 and 385. Among University Hospitals' isolates, no relationship was found between infection site and adherence.

### Biofilm Production

**Figure 4:**
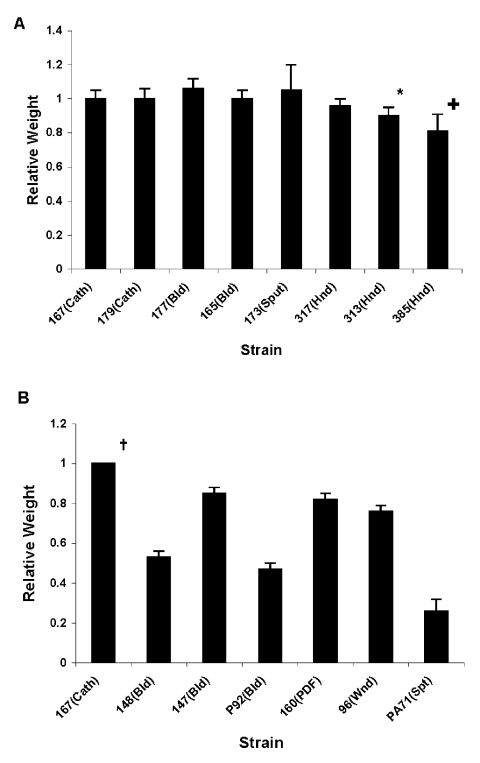
Relative dry weight of biofilms formed by *Candida parapsilosis* clinical isolates. Panel A shows relative dry weight of the *C. parapsilosis* strains from outbreak investigations by the Centers for Disease Control and Prevention from various culture sources. Results were normalized to control *C. parapsilosis* strain 167, which was taken as 100%. Each result is representative of at least two experiments. Error bars represent standard deviation. *p = 0.001 and p < 0.001 for dry weight comparison of 167 vs. 313 and 385, respectively. Panel B shows relative dry weight of the University Hospitals' *C. parapsilosis* strains from various culture sources, also compared to strain 167. p < 0.0005 for comparison of dry weight values of 167 vs. all others. (For details of methods used, see text.) Cath, catheter; Bld, bloodstream; Spt, sputum; Hnd, hand; Wnd, wound; Pdf, peritoneal dialysis fluid.

We first determined the ability of the *C. parapsilosis* outbreak isolates to form biofilm. All isolates of the same clone (strains 167, 165, 177, 179, 173, and 317) showed a similar pattern of biofilm formation by dry weight (Figure 4A), which suggests that biofilm formation by these isolates is consistent and not site-induced. Except for the unrelated environmental strains 313 and 385, biofilm formation was not significantly different for the various outbreak isolates (p > 0.05, when compared with strain 167). However, noninvasive strains 313 and 385 produced significantly less biofilm when measured by dry weight (compared with strain 167; p < 0.001 for 385 and p = 0.001 for 313).

Figure 4B shows a comparison of biofilm formation by the *C. parapsilosis* outbreak clone with isolates obtained from our University Hospitals' collection, including specimens from different body sites. The outbreak strain 167 produced more biofilm than the University Hospitals' isolates (p < 0.0005 for comparison of dry weight values of 167 vs. all others), which indicates that outbreak clonal isolates had a higher ability to form biofilms.

For both sets of isolates, biofilm production was examined when TPN solution was substituted for YNB medium because TPN promotes *C. parapsilosis* growth (*16*). TPN increased the dry weight of biofilms formed by the clonal strain (167) by up to 40% (p = 0.008). However, the pattern of results across strains was similar to YNB-based method (not shown).

### SAP Assays

**Figure 5:**
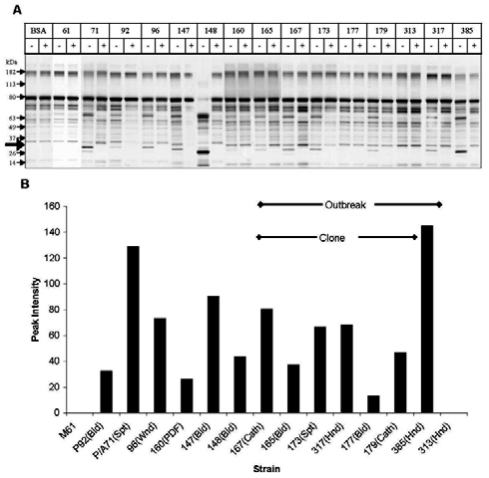
Secretory aspartic protease (SAP) expression by *Candida parapsilosis* clinical isolates. Panel A shows representative sodium dodecyl sulfate–polyacrylamide gel electrophoresis of various *C. parapsilosis* isolates. M, molecular weight marker lane; BSA, bovine serum albumin alone; other lanes show number of isolate; and +, supernatant plus protease inhibitor cocktail. Protease activity is evident from the appearance of lower molecular weight bands representing cleavage products. Thick arrow indicates the 20-kDa protein appearing after protease digestion. (For details of methods used see text.) Panel B shows densitometric scanning analysis of SAP activity. Strains 177 and 179 were included to demonstrate the heterogeneity in SAP production within the clonal strains. Cath, catheter; Bld, bloodstream; Spt, sputum; Hnd, hand; Wnd, wound; Pdf, peritoneal dialysis fluid.

We measured SAP production by assaying the ability of *C. parapsilosis* supernatant to hydrolyze BSA (*29*). A representative SDS-PAGE gel is shown in Figure 5A. The appearance of specific digestion products was noted when culture supernatant was added to BSA. Specifically, we observed the presence of a 20-kDa product, which did not appear when the reaction was carried out in presence of a protease inhibitor cocktail (Figure 5A, lanes marked "+"). This indicated that the 20-kDa band was a specific by-product of supernatant protease activity, present in all supernatants (except for strains M61 and 313). Analysis of the protease activity of culture supernatants was performed by densitometric scanning of the 20-kDa band. As seen in Figure 5B, the intensity of the 20-kDa product varied greatly between strains and within the outbreak clonal isolates, and no consistent pattern of protease activity was evident. These results were confirmed in multiple experiments.

### Phospholipase Assays

**Figure 6:**
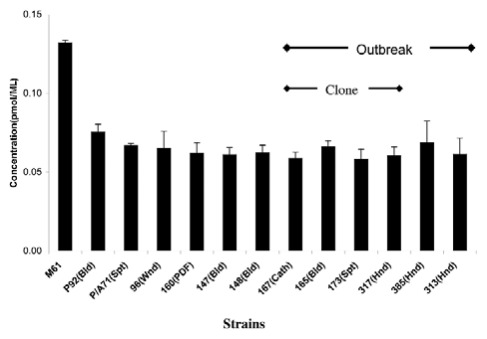
Phospholipase expression by *Candida parapsilosis* clinical isolates. Phospholipase expression as determined by the colorimetric method is shown. *C. albicans* train M61 was included as it is a known phospholipase producer. (For details of the methods used, see text.) Cath, catheter; Bld, bloodstream; Spt, sputum; Hnd, hand; Pdf, peritoneal dialysis fluid.

We determined the phospholipase activity of supernatants obtained from cultures of the different *C. parapsilosis* isolates, using a colorimetric assay (*30*). For comparative purposes, we included the phospholipase activity of *C. albicans* strain M61, since *C. albicans* is a known phospholipase producer. Although phospholipase activity varied among strains (Figure 6), no consistent differences were observed between sources (outbreak vs. University Hospitals), sites (e.g., blood vs. other), or clonality of isolates.

## Discussion

We studied a nosocomial outbreak of *C. parapsilosis* (*20*) and compared outbreak isolates with ones obtained from sporadic infections at our facility. Genetic analysis showed the invasive outbreak isolates (defined as being cultured from blood or catheter (*14**,**34*), as well as at least one hand isolate, were from the same clone. This finding agrees with results of previous epidemiologic studies of *C. parapsilosis* infections, which found predominant clonality (*10**,**35*). Since these clones were the same as environmental isolates, the outbreaks appear to have a nosocomial environmental origin. This conclusion also seems to be the case in the Mississippi outbreak. In contrast, the University Hospitals' strains were unrelated, indicating sporadic infection. Our results support assertions that molecular analysis is useful in investigating *C. parapsilosis* outbreaks (*36*).

Adhesion is likely a critical first step in yeast pathogenesis (*33*). This property might be expected for *C. parapsilosis*, which is thought to be acquired from exogenous sources, subsequently adheres to indwelling devices, and finally invades the host. However, our results agree with those of DeBernardis (*37*), who found no difference in adhesion between invasive and skin isolates of *C. albicans*. Other studies on *C. parapsilosis* adherence, in fact, showed an inverse relationship between invasiveness and adherence (*38*).

The clonal outbreak isolates produced more biofilm than either the unrelated environmental strains, or the University Hospitals' specimens. These results suggest that biofilm formation is an important component of an outbreak strain's ability to cause infection. Although previous studies of C. albicans (*14*) and C. parapsilosis (*16**,**39*) have suggested that bloodstream isolates produce more biofilm, further in vivo and clinical studies are needed to confirm these findings. If increased production of biofilm by outbreak isolates can be confirmed, this finding may point to a strategy for determining the significance of C. parapsilosis clinical isolates. Finally, our results confirm earlier work, suggesting that TPN promotes the development of C. parapsilosis biofilms (*39*).

Although secreted enzymes are likely virulence factors for all yeast species, patterns of expression vary across species. This variation may be exemplified by SAP phenotypes. Although SAPs may play a role in C. albicans adhesion (*40*), they are not important for the primary mode of invasion (through gut mucosa) because knockout strains do not display attenuated virulence (*19*). However, SAP may be important for pathogenesis at other sites or stages of infection. Regarding C. parapsilosis, DeBernardis et al. (*37*) found an inverse relationship with invasiveness. Our findings do not support the concept that SAP expression is a critical virulence factor for C. parapsilosis. The variability of our results even across a single clone also raises a larger question of the appropriateness of characterizing SAP expression as a stable experimental pathogenic factor in C. parapsilosis. Although previous work suggested that SAP expression is stable for a given isolate (*41*), wide variations exist in relevant mRNA production over fairly short periods (*42*).

Phospholipases are important virulence factors for C. albicans (*18*) but have not been well studied in C. parapsilosis. Although the isolates examined in this article produced phospholipase, no correlation was found between phospholipase activity and site of infection or other virulence factors. Although one article described phospholipase B and protease activity in a few strains of C. parapsilosis (*43*), ours is the first study to conduct a more detailed examination of phospholipase behavior in this species.

Our results also show no apparent correlations across the multiple putative virulence factors studied. This result agrees with Branchini's examination of genotypic variation and slime production in C. parapsilosis (*44*). Other studies that have reported correlation in expression of multiple virulence factors have been for C. albicans (*45*), of uncertain relevance to C. parapsilosis.

Although we did not find significant associations between most of the virulence factors and clinical pathologic changes, these results do not mean that these factors are unimportant. Rather, the results suggest that they are not critical to clinical outbreaks. Since all isolates expressed some degree of adhesion (except for hand isolates 313 and 385), SAP, and phospholipase B activity, these phenotypes may be necessary but not sufficient prerequisites for infection. Our results highlight the importance of using outbreak isolates (rather than those from sporadic infections or laboratory stocks) in studies of Candida virulence. Definitive analysis of the role of virulence factors will require further genetic analysis and in vivo models examining the behavior of knockout mutants of C. parapsilosis.

This study has some limitations. First, the number of isolates is small. Given the nature of *C. parapsilosis* infections and outbreaks, a higher *N* could not be expected, and the outbreak is especially well-characterized (*20*). Previous work has drawn conclusions from as few as one isolate or from less-characterized groups of isolates (*16*). Second, genetic analysis of *C. parapsilosis* that uses Cp3-13 fingerprinting may have limitations. Third, our SAP assay is unable to determine the relative contributions of different members of the gene family, of which there are at least 10 (*46*). Such analysis is beyond the scope of this paper. Fourth, similar limitations exist regarding characterization of phospholipase activity, as *Candida* expresses multiple phospholipases (*18*). Phospholipase expression varies with environmental conditions (*47*); however, we performed experiments under standardized conditions, physiologic temperature, and using glucose containing solutions (*14*).

In conclusion, the genotypic pattern of this C. parapsilosis outbreak suggests a clonal outbreak, likely arising from an environmental source and distinct from sporadic infection. The outbreak clone produced more biofilm than all other strains. No clear relationships were apparent for the other putative virulence factors, which suggests that they are not critical to outbreak behavior. Further genetic and in vivo studies are required to confirm these findings. Future analysis of virulence mechanisms likely needs to use outbreak strains, as well as taking into account the interplay of organism, host, and environment.
